# Relationship between the face scale for rating of perceived exertion and physiological parameters in older adults and patients with atrial fibrillation

**DOI:** 10.14814/phy2.14759

**Published:** 2021-03-02

**Authors:** Satoshi Nashimoto, Shinichiro Morishita, Susumu Iida, Kazuki Hotta, Atsuhiro Tsubaki

**Affiliations:** ^1^ Department of Rehabilitation Niigata Medical Center Niigata‐city, Niigata Japan; ^2^ Institute for Human Movement and Medical Sciences Niigata University of Health and Welfare Niigata‐city, Niigata Japan

**Keywords:** aerobic threshold, atrial fibrillation, cardiopulmonary exercise test, elderly, Face Scale, physiological parameters

## Abstract

**Background:**

The Borg scale is used to determine exercise intensity in rehabilitation but can be difficult for older adults to understand. By contrast, face scale that are used to evaluate pain are much easier to understand thanks to the inclusion of illustrations. On the other hand, the prevalence of atrial fibrillation (AF) increases with age. This study aimed to determine the validity of the face scale for rating perceived exertion (RPE‐face scale) in older adults and patients with AF during cardiopulmonary exercise test. Furthermore, the relationship between face scale and anaerobic threshold (AT) was also investigated.

**Methods:**

A total of 90 patients with sinus rhythm (SR) (74 men, 16 women) and 22 with AF were enrolled. Participants’ responses were recorded using the RPE‐face scale and compared with exercise intensity, heart rate, oxygen uptake, and minute ventilation during the exercise test. We determined the AT by the V‐slope method.

**Results:**

Correlations between RPE‐face scale and physiological parameters were significantly positive for men with SR and women with SR and AF. However, differences in the correlation coefficient between age and SR or AF were not statistically significant. The cutoff value for AT of the RPE‐face scale was “4,” showing high sensitivity and specificity.

**Conclusions:**

The RPE‐face scale can be used to determine the intensity of physical exercise, unaffected by age, gender, SR, or AF.

## Graphical abstract

- There were significant positive correlations between the face scale for rating perceived exertion (RPE‐face scale) and exercise intensity, heart rate, oxygen uptake, and minute ventilation. There was no statistically significant differences in the correlation coefficient between age and SR or AF.
- The cutoff value for AT of the RPE‐face scale was “4” and it showed high sensitivity and specificity.
- The RPE‐face scale can be used to determine the intensity of physical exercise equivalent to AT unaffected by age, gender, SR, or AF.

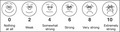

## 1 INTRODUCTION

Studies have shown that total daily physical activity levels are associated with cardiovascular death (Dorn et al., 1999; Inoue et al., 2008; Noda et al., 2005), with regular exercise known to be effective for the secondary prevention after myocardial infarction (Bäck et al., 2013; Clark et al., 2005; Janssen & Jolliffe, 2006; Leon et al., 2005; Taylor et al., 2004). Physical activity is also an independent factor for the prognosis of patients with heart failure (Izawa et al., 2013). Therefore, it is important that patients with heart disease continue to exercise regularly.

Exercise intensity is typically measured using either the heart rate (HR) or the rating of perceived exertion (RPE) (Borg, 1982). However, each method has problems when implemented with older adults. For example, HR can be unreliable in the presence of certain medications (Eston & Connolly, 1996) or arrhythmias (Borg & Dahlstrom, 1962). The most widely used RPE tool is the Borg scale, which has been shown to correlate with the HR and maximum oxygen uptake (Borg, 1998; Mihevic, 1981; Riebe et al., 2017). Although the Borg scale is usually reliable, children may have difficulty understanding it (Chen et al., 2017). Even in elderly people, correlation coefficients between the Borg scale and HR and maximum oxygen uptake are lower than those in young adults (Luana et al., 2020; Pak‐Kwong et al., 2015). Many scales have been developed for use by children (Chen et al., 2017; Groslambert et al., 2001; Quinart et al., 2016; Roemmich et al., 2006), yet there are few for elderly, especially those with morbidities. We, therefore, adapted a face scale that could be used to evaluate exercise levels (Morishita et al., 2018).

Face scale is often used in the evaluation of pain (Hockenberry et al., 2002). A notable example is the Wong‐Baker FACES Pain Rating Scale, which consists of a set of six faces that express different levels of distress. This scale is easy to understand because it is expressed as illustrations, making it useful for children or older adults with communication or cognitive issues (Bieri et al., 1990; Herr et al., 1998; Lewko et al., 2009; Wong & Baker, 1988). We previously validated a modified version of the FACES scale for use as an exercise intensity evaluation index. Our findings indicated that there was a significant correlation between the modified face scale (face scale for rating of perceived exertion, RPE‐face scale), HR, exercise load, and oxygen uptake (VO_2_) in healthy college students (Morishita, Tsubaki, et al., 2018). However, we did not determine whether it was suitable for use with older adults or patients suffering from morbidity.

In contrast, the prevalence of atrial fibrillation (AF) increases with age. Research has shown that the prevalence of AF is 2.3%–4.0% among those aged 65–70 years and 7.3%–15.4% in those aged ≥80 years (Furberg et al., 1994; Go et al., 2001; Heeringa et al., 2006; Majeed et al., 2001; Wolf et al., 1991). Considering population aging, the number of patients is expected to increase (Go et al., 2001). A case report has been published on RPE in AF cases (Borg & Dahlstrom, 1962) but there are few studies. The effect of exercise should also be considered in patients with AF (Hegbom et al., 2006; Mertens & Kavanagh, 1996), and the validity of RPE in patients with AF should be examined.

In this study, we aimed to determine the validity of the RPE‐face scale in older adults and patients with AF during cardiopulmonary exercise testing. In addition, anaerobic threshold point (AT) is one of the criteria used for prescribing exercise in patients with heart disease (Katch et al., 1978; Kindermann et al., 1979), and hence, its relationship with the RPE‐face scale is also investigated.

## 2 MATERIALS AND METHODS

### 2.1 Study design

The study was approved by the Ethics Committee of Niigata Medical Center (approval no. 2018‐04) and the Ethics Committee of Niigata University Health and Welfare Graduate School of Medicine (approval no. 17956‐180313). Written informed consent to participate in the study was obtained from all patients.

This was a prospective observational study of patients with AF who were referred to Niigata Medical Center from June 2018 to March 2019.

**FIGURE 1:**
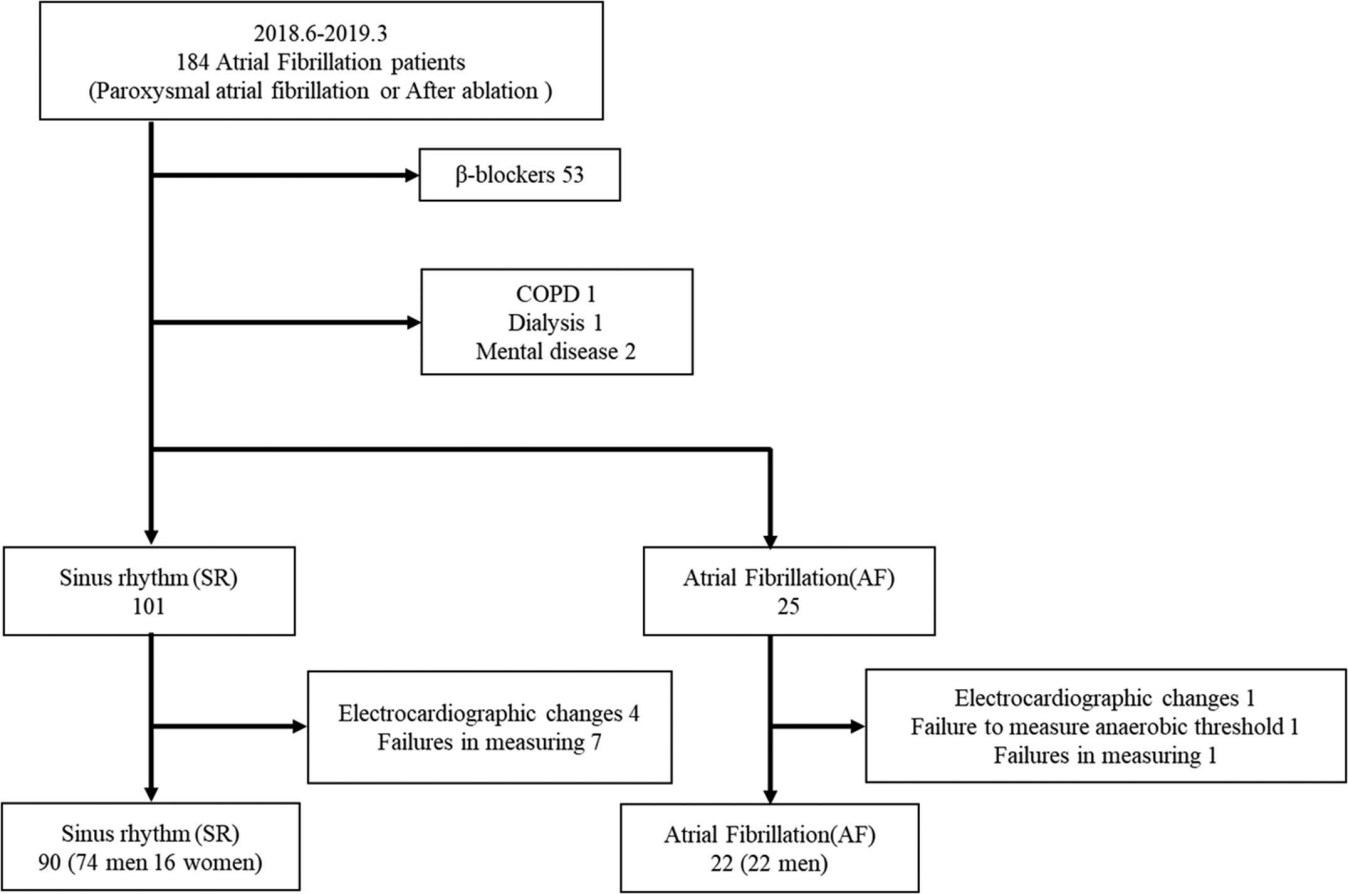
Flowchart of the patient recruitment process

We identified 184 patients with AF during the study period; of these, we enrolled 92 men and 20 women in sinus rhythm (SR). Patients aged ≥20 years who were referred to our hospital for treatment of AF were included in the study. Patients were excluded if they used beta‐blockers; if they had cardiac pacemakers or received dialysis; or if they were diagnosed with COPD or mental disease. In addition, electrocardiographic changes appeared during the exercise test in five patients (SR to paroxysmal AF [*n* = 1], SR to paroxysmal atrial flutter [*n* = 2] and to paroxysmal atrial tachycardia [*n* = 1], AF to SR [*n* = 1]). It was difficult to measure the AT in one patient, and measurement failed in eight patients. Thus, 90 patients with SR (diagnosis of paroxysmal AF but SR during the exercise test, 74 men and 16 women) and 22 patients with AF (22 men) were analyzed (Figure 1).

### 2.2 Clinical variables

Peripheral blood samples were collected for the measurement of C‐reactive protein level, hemoglobin (Hb) level, brain natriuretic peptide (BNP) level, estimated glomerular filtration rate (eGFR), and glycosylated hemoglobin (HbA1c) level, in addition to evaluating the left ventricular ejection fraction (LVEF) in the echocardiography. We investigated the complications of diabetes, hypertension, and smoking. All patients with diabetes and hypertension were on medication and well controlled.

### 2.3 Face scale for rating of perceived exertion, RPE‐face scale

**FIGURE 2:**
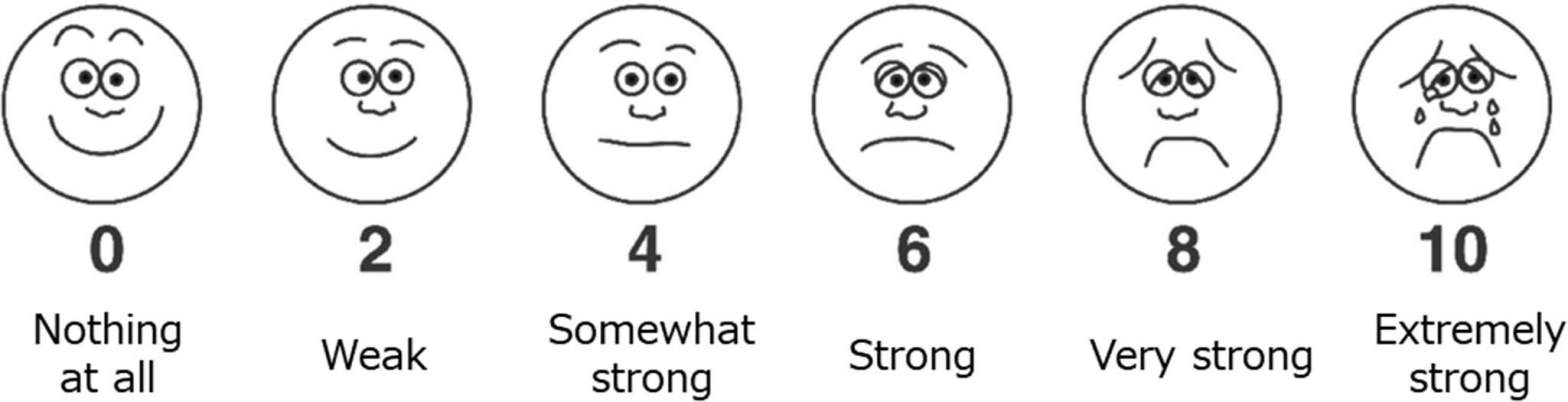
The face scale for rating of perceived exertion; RPE‐face scale

A modified version of the face scale (RPE‐face scale) was used (Hockenberry et al., 2002; Morishita, Tsubaki, et al., 2018), as shown in Figure 2. This presents a set of six faces that express various levels of distress in a format that is easy to understand. The scale score ranges from 0 to 10 and includes numbers, face illustrations, and verbal expressions. Participants were asked to choose what best described their feelings at a given assessment point. At 1 min intervals during the exercise tests, participants were asked “How hard do you feel you are working?” and to rate their RPE on the RPE‐face scale by pointing.

### 2.4 Cardiopulmonary exercise tests

We measured HR, VO_2_, intensity of exercise (WR), and ventilation (VE) by cardiopulmonary exercise tests, using stationary bicycles (Wellbike BE‐260; FUKUDA DENSHI, Tokyo, Japan). Participants began exercise tests with a 3‐min rest in a sitting position before the warm‐up phase of 3 min cycling at 10 W. They then cycled under a 10 W/min ramp load test until maximum fatigue was achieved. All subjects were instructed to maintain a cadence of 50 rotations per minute (rpm) throughout the cardiopulmonary exercise test until they reached exhaustion. If a subject could not maintain at least 50 rpm, the cardiopulmonary exercise test was discontinued. After test completion, a 3‐min cool‐down period of cycling at 10 W was completed by each participant.

During the cardiopulmonary exercise test, HR was recorded by a 12‐lead surface electrocardiogram (Stress Test System ML‐6500; FUKUDA DENSHI, Tokyo, Japan). VO_2_, VE, and carbon dioxide emissions were measured by an exhaled gas analyzer (Cpex‐1; Inter Reha, Tokyo, Japan). Each parameter was recorded every minute when the patients were asked to rate their RPE on the RPE‐face scale. Additionally, we determined the anaerobic thresholds by the V‐slope method during the cardiopulmonary exercise test, and we calculated R(VCO_2_/ VO_2_) at maximum load, VE/VCO_2_, Peak VO_2_/HR, and VO_2_/WR. We measured systolic and diastolic blood pressure (sBP, dBP) at the start of testing.

### 2.5 Statistical analysis

All data analyses were performed in R (version 2.6.1) and a value of *p* < 0.05 was considered statistically significant. Correlation analyses were then performed based on gender group, the individual, and age, and are reported with 95% CI.

First, the patient characteristics and clinical variance were examined using the Shapiro–Wilk test and Levene's test, respectively. Two‐sample *t*‐test was used to analyze the data with normal distribution and equal variance. The Welch test was used to analyze the data with normal distribution and without equal variance, and the Mann–Whitney *U* test was used to analyze the data without normal distribution. We compared the variables between men and women, between those aged ≥65 years and those aged <65 years, and between the SR and AF.

Second, Spearman's rank correlation coefficients (ρ) were calculated to evaluate the correlation between the RPE‐face scale and the HR, VO_2_, WR, and VE each minute during the exercise test, and comparing the correlation coefficient with those aged ≥65 years and those aged <65 years by gender and cardiac rhythm. Correlations between parameters were assessed using bivariate simple correlation analysis, with Spearman's rank correlation coefficient for non‐parametric values.

Third, Spearman's rank correlation coefficients (ρ) were calculated to evaluate the correlation between the RPE‐face scale and the HR, VO_2_, WR, and VE each minute during the exercise test for individuals.

Fourth, receiver operating characteristic curve analysis was performed to determine the cutoff value of the RPE‐face scale for AT prediction. We determined the cutoff value, sensitivity, specificity, and area under the curve (AUC) of the RPE‐face scale.

Finally, for subgroup analysis, Spearman's rank correlation coefficient (ρ) was calculated to evaluate the correlation between the RPE‐face scale and the HR, VO_2_, WR, and VE each minute during the exercise test, except for smokers, those with diabetes, and those with hypertension.

## 3 RESULTS

### 3.1 Patient characteristics and clinical variance

Table 1 shows the participant characteristics and clinical variables; Table 2 shows the CPX results. There were 37 men aged ≥65 years and 37 aged <65 years old. There were 10 women aged ≥65 years and 6 aged <65 years. Moreover, there were 22 patients with AF.

**TABLE 1:** Participant characteristics and clinical variable

	Sinus rhythm Men			Sinus rhythm Women			Atrial fibrillation, *n* = 22
	All *n* = 74	≥65 years old *n* = 37	<65 years old *n* = 37	All *n* = 16	≥65 years old *n* = 10	<65 years old *n* = 6	
Age, years	63.0 ± 10.0	70.6 ± 5.0	55.5 ± 7.9**	66.3 ± 6.4	70.3 ± 3.6	59.7 ± 3.7 ^**^	60.0 ± 8.1
Height, cm	170.5 ± 5.5	168.8 ± 5.7	172.3 ± 4.7	155.9 ± 5.2	154.3 ± 5.0	158.7 ± 4.9	171.6 ± 7.1
Body weight, kg	71.0 ± 9.1	68.8 ± 7.1	73.3 ± 10.3	57.4 ± 6.7	56.3 ± 6.3	59.1 ± 7.5	75.9 ± 12.0
Body mass index, kg/m^2^	24.4 ± 2.8	24.1 ± 2.3	24.7 ± 3.2	23.7 ± 3.4	23.9 ± 3.8	23.5 ± 2.8	25.8 ± 3.9
Diagnosis							
Paroxysmal atrial fibrillation	33	18	15		4	5	20
After ablation (atrial fibrillation)	41	19	22		6	1	2
Result of blood test							
CRP, mg/dl	0.10 ± 0.15	0.13 ± 0.18	0.07 ± 0.08	0.14 ± 0.14	0.15 ± 0.11	0.12 ± 0.19	0.07 ± 0.06
Hb, g/dl	14.5 ± 1.0	14.2 ± 1.2	14.8 ± 0.8*	13.4 ± 0.98	13.2 ± 0.9	13.8 ± 1.1	15.3 ± 2.1^‡^
BNP, pg/ml	39.3 ± 53.0	55.8 ± 67.2	22.7 ± 25.1**	38.2 ± 43.6	46.4 ± 53.7	24.6 ± 12.2	80.9 ± 56.1^‡^
eGFR, ml/min	65.7 ± 13.3	63.8 ± 13.7	67.6 ± 12.9	89.7 ± 19.4	85.0 ± 23.1	97.4 ± 7.4	64.1 ± 12.8
HbA1c, %	5.9 ± 0.6	5.9 ± 0.6	5.8 ± 0.6	5.8 ± 0.5	5.9 ± 0.6	5.6 ± 0.3	6.5 ± 2.3
LVEF, %	65.2 ± 7.6	66.6 ± 5.9	63.9 ± 8.8	68.1 ± 4.8	68.9 ± 5.8	66.7 ± 2.0	64.2 ± 4.7
Diabetes mellitus, number, %	12, 16.2%	10, 27.0%	2, 5.4%*	2, 12.5	2, 20%	0, 0%	5, 22.7%
Hypertension, number, %	36, 48.6%	24, 64.9%	12, 32.4%*	5, 31.3%	4, 40%	1, 16.7%	13, 59.0%
Smoker, number, %	46, 61.3%	22, 59.5%	24, 64.9%	3, 18.8%	0, 0%	3, 50% ^†^	16, 72.7%

Note

Reported as mean ± SD or number, %.

Abbreviations: CRP, C‐reactive protein level; Hb, hemoglobin level; BNP, brain natriuretic peptide level; eGFR, estimated glomerular filtration rate; HbA1c, glycosylated hemoglobin; LVEF, left ventricular ejection fraction.

* Represents a significant (*p* < 0.05) difference between the men group ≥65 years old and group <65 years old.

** Represents a significant (*p* < 0.01) difference between the men group ≥65 years old and group <65 years old.

† Represents a significant (*p* < 0.05) difference between the women group ≥65 years old and group <65 years old.

†† Represents a significant (*p* < 0.01) difference between the women group ≥65 years old and group <65 years old.

‡ Represents a significant (*p* < 0.01) difference between the men group sinus rhythm and group atrial fibrillation.

**TABLE 2:** Parameters of cardiopulmonary exercise testing

	Sinus rhythm Men			Sinus rhythm Women			Atrial fibrillation *n* = 22
	All *n* = 74	≥65 years old *n* = 37	<65 years old *n* = 37	All *n* = 16	≥65 years old *n* = 10	<65 years old *n* = 6	
Rest							
RPE‐face scale	0.1 ± 0.6	0.1 ± 0.5	0.1 ± 0.7	0.3 ± 0.7	0.2 ± 0.6	0.3 ± 0.8	0.09 ± 0.4
HR, beats/min	77.1 ± 13.9	76.0 ± 15.1	78.4 ± 12.8	77.7 ± 14.3	79.1 ± 15.6	75.3 ± 12.7	87.6 ± 20.0^‡^
VO_2_, ml/min/kg	3.9 ± 0.8	3.9 ± 0.9	4.0 ± 0.9	3.7 ± 0.7	3.5 ± 0.6	4.0 ± 0.9	3.7 ± 1.0
VE, L/min	11.7 ± 2.7	11.8 ± 2.7	11.6 ± 2.6	9.1 ± 1.6	8.7 ± 1.3	9.8 ± 2.0	12.1 ± 2.7
WR, watts	0	0	0	0	0	0	0
sBP, mmHg	133.8 ± 18.7	140.5 ± 18.7	127.2 ± 16.4**	136.0 ± 22.1	139.3 ± 21.3	130.5 ± 24.4	122.8 ± 18.7^‡^
dBP, mmHg	84.2 ± 10.3	81.5 ± 10.5	82.5 ± 10.2	83.6 ± 16.5	86.6 ± 18.8	78.7 ± 11.6	84.2 ± 15.1
Anaerobic threshold							
RPE‐face scale	2.9 ± 2.0	3.1 ± 2.0	2.7 ± 1.9	2.6 ± 1.8	2.6 ± 1.9	2.7 ± 1.6	3.8 ± 1.5^‡^
HR, beats/min	96.3 ± 15.4	95.9 ± 18.5	96.8 ± 11.8	94.3 ± 16.3	96.2 ± 16.9	91.0 ± 16.1	116.9 ± 27.1^‡^
VO_2_, ml/min/kg	12.8 ± 3.2	12.3 ± 3.4	13.3 ± 3.0	9.8 ± 1.7	9.6 ± 1.8	10.2 ± 1.6	12.7 ± 3.6
VE, L/min	32.7 ± 8.7	33.4 ± 8.7	31.9 ± 8.7	22.6 ± 6.1	21.8 ± 7.2	24.1 ± 3.7	33.3 ± 6.0
WR, watts	60.1 ± 12.7	56.2 ± 11.6	64.0 ± 12.7**	41.3 ± 6.0	41.5 ± 7.1	41.0 ± 4.0	69.9 ± 26.7
End of test (maximum)							
RPE‐face scale	8.3 ± 2.0	7.9 ± 2.3	8.7 ± 1.6	7.8 ± 1.4	7.4 ± 1.3	8.3 ± 1.5	9.4 ± 0.8^**^
HR, beats/min	127.6 ± 21.2	126.9 ± 24.6	128.4 ± 17.6	118.1 ± 21.4	114.3 ± 18.6	124.3 ± 26.0	151.1 ± 30.9^**^
VO_2_, ml/min/kg	19.9 ± 4.7	18.6 ± 4.2	21.2 ± 4.8*	14.6 ± 3.2	13.4 ± 2.4	16.7 ± 3.6^†^	18.7 ± 5.2
VE, L/min	55.7 ± 17.0	55.1 ± 17.6	56.2 ± 16.5	35.8 ± 7.9	33.6 ± 6.9	39.4 ± 8.7	57.7 ± 16.8
WR, watts	117.7 ± 26.1	110.0 ± 27.2	125.5 ± 22.9**	71.8 ± 14.5	68.5 ± 11.7	77.3 ± 18.1	128.2 ± 42.0
R	1.4 ± 0.1	1.4 ± 0.1	1.3 ± 0.1*	1.3 ± 0.2	1.3 ± 0.1	1.4 ± 0.2	1.3 ± 0.1
VE/VCO_2_	23.8 ± 4.1	25.2 ± 3.9	22.4 ± 3.9**	26.3 ± 5.8	27.0 ± 3.9	25.2 ± 8.4	24.0 ± 4.7
PeakVO_2_/HR, ml/beats	11.1 ± 2.6	10.2 ± 2.2	12.0 ± 2.7**	7.3 ± 1.9	6.8 ± 2.0	8.0 ± 1.5	9.6 ± 2.8^‡^
VO_2_/WR, ml/min/watts	9.5 ± 1.5	9.1 ± 1.7	9.9 ± 1.1**	7.7 ± 1.3	7.3 ± 1.0	8.4 ± 1.6	9.2 ± 1.6

Note

Reported as mean ± SD.

Abbreviations: RPE‐face scale, Face scale for rating of perceived exertion; HR, heart rate; VO_2_, oxygen uptake; VE, ventilation; WR, work rate; R, gas Exchange ratio of CO_2_ output to O_2_ uptake; VCO_2_, carbon dioxide output; sBP, systolic blood pressure; dBP, diastolic blood pressure.

* Represents a significant (*p* < 0.05) difference between the men group ≥65 years old and group <65 years old.

** Represents a significant (*p* < 0.01) difference between the men group ≥65 years old and group <65 years old.

† Represents a significant (*p* < 0.05) difference between the women group ≥65 years old and group <65 years old.

‡ Represents a significant (*p* < 0.05) difference between the men group sinus rhythm and group atrial fibrillation.

‡‡ Represents a significant (*p* < 0.01) difference between the men group sinus rhythm and group atrial fibrillation.

In men aged >65 years, the Hb, WR at AT, maximum VO_2_ and WR, peak VO_2_/HR, and VO_2_/WR were significantly lower, whereas BNP, R, and sBP, VE/VCO_2_ were significantly greater than those in men aged <65 years. In women, smoking rates, and maximum VO_2_ were significantly lower in those aged ≥65 years. In AF, Hb, BNP, HR of rest, AT, and maximum, maximum RPE‐face scale were significantly greater than SR of men. Peak VO_2_/HR was significantly lower than SR of men.

### 3.2 Relationship between RPE‐face scale and physiological parameters

There were significant positive correlations by gender between the RPE‐face scale and the exercise test parameters. The correlation coefficient tended to decrease with increasing age, but there was no difference in the correlation coefficient between over the age of 65 years and older group and under the age of 65 years old group. There were no statistically significant differences between SR men (*n* = 74) and AF men (*n* = 22) (Table 3).

**TABLE 3:** Correlation analyses at a group level and correlation coefficient comparison

	Sinus rhythm Men					Sinus rhythm Women				Atrial fibrillation *n* = 22	*p* value^‡^
	All *n* = 74	≥65 years old *n* = 37	<65 years old *n* = 37	*p*‐value*	All *n* = 16		≥65 years old *n* = 10	<65 years old *n* = 6	*p*‐value^†^		
HR	0.58	0.51	0.65	0.36	0.45		0.54	0.32	0.70	0.63	0.76
VO_2_	0.76	0.74	0.77	0.78	0.63		0.55	0.74	0.64	0.82	0.53
VE	0.72	0.71	0.73	0.87	0.74		0.67	0.84	0.56	0.87	0.10
WR	0.81	0.78	0.84	0.48	0.75		0.74	0.76	0.94	0.85	0.62

Abbreviations: HR, heart rate; VO_2_, oxygen uptake; VE, ventilation; WR, work rate.

* Represents a *p*‐value in the correlation coefficient comparison between the men group ≥65 years old and group <65 years old.

† Represents a *p*‐value in the correlation coefficient comparison between the women group ≥65 years old and group <65 years old.

‡ Represents a *p*‐value in the correlation coefficient comparison between the men group sinus rhythm and group atrial fibrillation.

In addition, all correlation coefficients were significant for individuals (*p* < 0.05), and many cases showed a strong correlation of ≥0.8 at this level (Table 4).

**TABLE 4:** Correlation analyses at an individual level

	Sinus rhythm Men		Sinus rhythm Women		Atrial fibrillation *n* = 22
	≥65 years old *n* = 37	<65 years old *n* = 37	≥65 years old *n* = 10	<65 years old *n* = 6	
HR	0.94 (0.88–0.97)	0.93 (0.91–0.96)	0.92 (0.88–0.95)	0.90 (0.80–0.95)	0.94 (0.89–0.96)
VO_2_	0.93 (0.85–0.97)	0.93 (0.89–0.96)	0.92 (0.87–0.95)	0.91 (0.81–0.94)	0.94 (0.92–0.96)
VE	0.94 (0.85–0.97)	0.93 (0.90–0.96)	0.92 (0.85–0.94)	0.94 (0.91–0.95)	0.95 (0.93–0.96)
WR	0.96 (0.88–0.97)	0.96 (0.92–0.97)	0.93 (0.90–0.96)	0.96 (0.95–0.97)	0.97 (0.96–0.97)

Results are shown as median (25%–75% value). Correlation coefficients for all individuals were significant (*p* < 0.05).

Abbreviations: HR, heart rate; VO_2_, oxygen uptake; VE, ventilation; WR, work rate.

### 3.3 Cutoff value of the RPE‐face scale for AT

On the RPE‐face scale, the number of records below AT were 314 and the number of records above AT were 194 in men aged ≥65 years, men aged ≤65 years 337 and 222, women aged ≥65 years 67 and 29, women aged ≤65 years 39 and 24, AF 209 and139.

**FIGURE 3:**
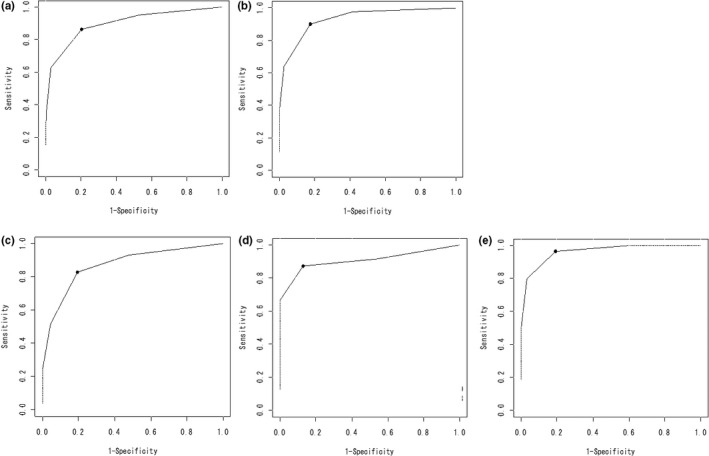
Receiver operating characteristic curve analysis to determine the cutoff value of the RPE‐face scale for it to predict anaerobic threshold. a, men over the age of 65 years and older group. b, men under the age of 65 years old group. c, women over the age of 65 years and older group. d, women under the age of 65 years old group. e, AF group. Note: AF, atrial fibrillation

In men aged ≥65 years, the cutoff value for the RPE‐face scale score was 4; the sensitivity of the scale was 86.6% (95% CI: 81.1%–90.7%), the specificity was 79.6% (95% CI: 74.8%–83.7%), and the AUC was 0.90 (*p* < 0.01). In men aged <65 years, the cutoff value for the RPE‐face scale was 4; the sensitivity of the scale was 90.0% (95% CI: 85.5%–93.3%), the specificity was 82.5% (95% CI: 78.1%–86.2%), and the AUC was 0.93 (*p* < 0.01). In women aged ≥65 years, the cutoff value for the RPE‐face scale was 4, the sensitivity was 82.8% (95% CI: 65.5%–92.4%), the specificity was 80.6% (95% CI: 69.6%–88.3%), and the AUC was 0.87 (*p* < 0.01). In women aged <65 years, the cutoff value for the RPE‐face scale was 4, the sensitivity was 87.5% (95% CI: 69.0%–95.7%), the specificity was 87.2% (95% CI: 73.3%–94.4%), and the AUC was 0.91 (*p* < 0.01). In those with AF, the cutoff value of the RPE‐face scale was 4, the sensitivity was 96.4% (95% CI: 91.9%–98.5%), the specificity was 80.9% (95% CI: 75.0%–85.6%), and the AUC was 0.96 (*p* < 0.01) (Figure 3).

### 3.4 Relationship between RPE‐face scale and physiological parameters excluding smokers, participants with diabetes, and participants with hypertension

Table 5 shows the participant characteristics and CPX results, excluding smokers, participants with diabetes, and participants with hypertension. There were 6 men aged ≥65 years and 10 aged <65 years old. There were 5 women aged ≥65 years and 2 aged <65 years. Moreover, there were 4 patients with AF.

**TABLE 5:** Participant characteristics and parameters of cardiopulmonary exercise testing excluding smokers, participants with diabetes, and participants with hypertension

	Sinus rhythm Men			Sinus rhythm Women			Atrial fibrillation *n* = 4
	All *n* = 16	≥65 years old *n* = 6	<65 years old *n* = 10	All *n* = 7	≥65 years old *n* = 5	<65 years old *n* = 2	
Age, years	56.7 ± 12.5	68.8 ± 4.5	49.2 ± 9.5	67.7 ± 7.3	71.6 ± 3.4	58.0 ± 2.8	55.5 ± 7.5
Height, cm	169.5 ± 4.7	169.5 ± 7.0	169.4 ± 3.1	158.3 ± 3.9	157.3 ± 4.3	160.7 ± 0.2	169.0 ± 4.8
Body weight, kg	69.0 ± 9.3	69.4 ± 9.7	68.7 ± 9.5	54.1 ± 6.8	52.6 ± 6.8	57.8 ± 7.4	72.3 ± 6.3
Body mass index, kg/m^2^	24.0 ± 3.0	24.1 ± 2.0	24.0 ± 3.6	21.6 ± 3.1	21.4 ± 3.4	22.4 ± 3.0	25.3 ± 1.6
Rest							
RPE‐face scale	0	0	0	0.6 ± 1.0	0.4 ± 0.9	1.0 ± 1.4	0
HR, beats/min	81.3 ± 12.3	80.2 ± 16.6	82.0 ± 9.9	7.87 ± 16.3	81.0 ± 15.9	73.0 ± 22.6	89.5 ± 23.7
VO_2_, ml/min/kg	4.0 ± 0.6	3.9 ± 0.7	3.9 ± 0.6	3.9 ± 1.0	3.5 ± 0.8	5.0 ± 0.4	4.2 ± 0.8
VE, L/min	10.5 ± 2.1	11.4 ± 1.7	10.0 ± 2.2	9.3 ± 2.2	8.2 ± 1.5	11.9 ± 0.4	12.9 ± 2.3
WR, watts	0	0	0	0	0	0	0
sBP, mmHg	124.8 ± 14.4	128.2 ± 15.7	122.7 ± 14.0	129.3 ± 24.5	135.6 ± 26.4	113.5 ± 10.6	12.1.0 ± 24.3
dBP, mmHg	82.5 ± 11.0	83.2 ± 13.4	82.1 ± 10.0	84.1 ± 20.2	89.0 ± 22.0	72.0 ± 9.9	90.3 ± 9.3
Anaerobic threshold							
RPE‐face scale	3.3 ± 1.8	4.3 ± 2.0	2.6 ± 1.3	3.1 ± 1.6	3.2 ± 1.8	3.0 ± 1.4	4.5 ± 1.9
HR, beats/min	103.1 ± 16.7	107.3 ± 26.6	100.5 ± 7.1	97.3 ± 19.5	101.8 ± 17.9	86.0 ± 25.5	125.0 ± 39.4
VO_2_, ml/min/kg	11.6 ± 2.2	10.4 ± 2.0	12.3 ± 2.0	9.9 ± 2.0	9.1 ± 1.7	11.8 ± 1.6	14.7 ± 3.9
VE, L/min	27.8 ± 6.6	30.0 ± 6.5	26.6 ± 6.7	20.5 ± 6.0	18.3 ± 5.7	25.9 ± 2.5	34.3 ± 2.6
WR, watts	58.0 ± 6.9	52.7 ± 6.8	61.2 ± 4.7	41.6 ± 6.0	42.0 ± 4.8	40.5 ± 0.7	77.0 ± 28.9
End of test (maximum)							
RPE‐face scale	9.0 ± 1.3	9.7 ± 0.8	8.6 ± 1.3	7.1 ± 1.6	6.4 ± 0.9	9.0 ± 1.4	10
HR, beats/min	129.0 ± 18.5	124.8 ± 25.2	131.5 ± 14.1	122.9 ± 18.6	119.2 ± 21.3	132.0 ± 1.4	150.3 ± 33.0
VO_2_, ml/min/kg	19.9 ± 4.4	16.6 ± 3.7	21.8 ± 3.5	14.9 ± 4.3	12.7 ± 2.8	20.2 ± 0.8	23.9 ± 8.1
VE, L/min	48.8 ± 11.9	46.2 ± 12.7	50.3 ± 11.9	35.4 ± 11.5	29.8 ± 7.2	49.4 ± 6.6	74.2 ± 31.6
WR, watts	117.1 ± 22.8	103.2 ± 23.1	125.5 ± 19.0	71.1 ± 16.1	64.4 ± 10.8	88.0 ± 17.0	139.3 ± 49.3
R	1.3 ± 0.1	1.3 ± 0.1	1.3 ± 0.1	1.4 ± 0.2	1.3 ± 0.2	1.5 ± 0.2	1.4 ± 0.2
VE/ VCO_2_	22.2 ± 2.1	23.3 ± 1.3	21.5 ± 2.2	24.2 ± 5.2	26.8 ± 2.9	17.7 ± 3.7	22.5 ± 4.7
Peak VO_2_/HR, ml/beats	11.0 ± 3.2	9.4 ± 2.4	11.9 ± 3.4	6.7 ± 2.2	5.8 ± 1.8	8.9 ± 1.8	11.4 ± 1.7
VO_2_/WR, ml/min/watts	9.4 ± 1.2	8.6 ± 1.5	9.9 ± 0.9	7.9 ± 1.8	7.1 ± 1.0	9.9 ± 2.1	10.0 ± 1.5

Note.

Reported as mean ± SD.

Abbreviations: RPE‐face scale, face scale for rating of perceived exertion; HR, heart rate; VO_2_, oxygen uptake; VE, ventilation; WR, work rate; R, gas Exchange ratio of CO_2_ output to O_2_ uptake; VCO_2_, carbon dioxide output; sBP, systolic blood pressure; dBP, diastolic blood pressure.

There were significant positive correlations by gender between the RPE‐face scale and the physiological parameters (Table 6). The correlation coefficients tended to be higher than those that included smokers, participants with diabetes, and participants with hypertension.

**TABLE 6:** Correlation analyses at a group level excluding smokers, participants with diabetes, and participants with hypertension

	Sinus rhythm Men		Sinus rhythm Women		Atrial fibrillation *n* = 4
	≥65 years old *n* = 6	<65 years old *n* = 10	≥65 years old *n* = 5	<65 years old *n* = 2	
HR	0.54	0.79	0.54	0.54	0.79
VO_2_	0.88	0.83	0.88	0.82	0.85
VE	0.82	0.79	0.82	0.88	0.84
WR	0.91	0.90	0.91	0.91	0.85

Correlation coefficients for all individuals were significant (*p* < 0.05).

Abbreviations: HR, heart rate; VO_2_, oxygen uptake; VE, ventilation; WR, work rate.

## 4 DISCUSSION

In this study, the relationship between RPE‐face scale and various physiological parameters during a cardiopulmonary exercise test is investigated in older adults and patients with AF. The main findings of our study are as follows: (1) our previously modified face scale for RPE significantly correlates with HR, VO_2_, WR, and VE in older adults and patients with AF and (2) the cutoff value for the RPE‐face scale is “4,” showing high sensitivity and specificity. Thus, all things considered, the RPE‐face scale can be used to determine the physical exercise intensity, unaffected by age, gender, SR, or AF.

### 4.1 Clinical variance

The association between AF and heart failure (Santhanakrishnan et al., 2016) and chronic kidney disease (Alvaro et al., 2011) is well known, so we examined the cardiac and renal functions of the participants. With respect to the blood test data, although Hb tended to be low in the elderly subjects and BNP tended to be higher in elderly and AF patients, there were no outliers (Januzzi et al., 2005; Richards et al., 2013), no cases of anemia, and no patients with heart failure symptoms. In few patients, there was a decline in the LVEF and ventilation capacity (VE/VCO_2_). Based on the results of CPX, the average R was 1.3–1.4, and the maximum intensity could be applied.

### 4.2 Relationship between RPE‐face scale and physiological parameters

To date, the most widely used assessment tool for measuring RPE has been the Borg scale (Riebe et al., 2017), the validity of which has been evidenced by its high correlation with relevant physiological parameters. For example, the Borg scale has been shown to have a moderately high positive correlation between RPE and physiological variables such as HR, VO_2_, and VE (Pandolf, 1983; Swain et al., 1998). A meta‐analysis (Chen et al., 2002) further supported this correlation, reporting mean validity coefficients for the Borg scale with the HR, VO_2_, and VE of 0.62 (*n* = 3708), 0.63 (*n* = 332), and 0.61 (*n* = 357), respectively. Moreover, a recent cohort study stated that the correlation between HR and the Borg scale was 0.74 among 2560 large population (Scherr et al., 2013).

The correlation coefficients between the RPE‐face scale and the physiological parameters were moderate to high positive correlation in the present study among both men (0.58 for HR, 0.76 for VO_2_, 0.81 for WR, 0.72 for VE) and women (0.45 for HR, 0.63 for VO_2_ and 0.75 for WR, 0.74 for VE). It was also noteworthy that we found no difference in the correlation coefficient when grouping by age older or younger than 65 years and SR or AF. In earlier research, we showed correlations between the RPE‐face scale and these physiological measures of ≥0.8 in healthy college students (Morishita, Tsubaki, et al., 2018). Although the correlation coefficients among older adults were lower in this study especially in relation to HR. We consider that the influence of age on the HR response accompanying exercise was large (Brubaker & Kitzman, 2011). In the grouping of this study, compared with previous studies targeting students, there is a range of ages of the subjects, the variance of the maximum HR was large, and the correlation coefficient was small. Moreover, no details about exercise habits were collected in this study. Training reportedly affects the HR response (Paolillo et al., 2018), and it is possible that exercise habits affected the HR response. Group‐wise comparison of elderly patients showed that paying attention to the variation may be necessary. In AF patients, the HR response accompanying exercise is faster and higher due to a lower stroke volume (Paolillo et al., 2018). In the AF‐only group, the correlation between HR and RPE‐face scale score remained unchanged.

Many reports have examined the correlations between physiological parameters and various scales for RPE at a group level (Chen et al., 2017; Cleland et al., 2016; Groslambert et al., 2001; Morishita, Tsubaki, et al., 2018; Morishita, Wakasugi, et al., 2018; Penko et al., 2017; Quinart et al., 2016; Roemmich et al., 2006). However, when using this approach, there is a risk that the correlation coefficient will be lowered because individual affects the maximum value of the evaluation. In a meta‐analysis (Chen et al., 2002) of the relationship between RPE and physiological parameters, the authors reported that there was a negative correlation between the number of subjects and the resulting correlation coefficient. Methods for examining correlation in individuals were reported in one study for eight patients with Alzheimer's disease (Yu et al., 2015), and despite the sparsity of supporting reports. Although Mary et al. (1989) reported that correlations calculated from individuals overestimate the relationship between objective and subjective measures of exercise intensity, the method seems effective when dealing with data of patients with various backgrounds. Therefore, to supplement the analysis at the group level, we examined the correlation coefficient at the individual level. This revealed higher correlations of HR, VO_2_, WR, and VE with the RPE‐face scale (≥0.8 in most cases for both men and women). At least for individual, the RPE‐face scale benefits from being illustrated and easy to understand, and we anticipate that its use will be unaffected by gender and age, SR or AF.

### 4.3 Cutoff value of the RPE‐face scale for AT

Our results showed that the cutoff value for the RPE‐face scale was 4 “Somewhat Strong” the sensitivity was 82.8%–96.4%, and the specificity was 79.6%–87.2%. If the RPE‐face scale score during exercise is ≥4 (e.g., 6, 8, or 10), it is highly possible that the exercise intensity exceeded the AT. A Borg scale score of 13 indicated “Somewhat Hard” and was considered the AT (Purvis & Cureton, 1981; Scherr et al., 2013); in the RPE‐face scale, the same expression was the AT point. The AT point is important in exercise prescription because it suppresses the rise in lactic acid and catecholamine levels and can maintain exercise and the response of cardiac function with the exercise (Katch et al., 1978; Kindermann et al., 1979). The RPE‐face scale score can be used to prescribe an exercise intensity equivalent to the AT, irrespective of patient age and sex, and the presence of SR or AF.

### 4.4 Relationship between RPE‐face scale and physiological parameters excluding smokers, participants with diabetes, and participants with hypertension

The correlation coefficients between the RPE‐face scale and the physiological parameters were moderate to high positive correlation that tended to be higher than those that included smokers, participants with diabetes, and participants with hypertension. The effects on HR of smoking (Papathanasiou et al., 2013), diabetes (Sydó et al., 2016), and hypertension (Shen et al., 2020) have been reported. Although the number of participants in the present study was small, the involvement of these comorbidities in RPE could be possible.

### 4.5 Study limitations

There are several limitations of this study. First, only AF cases were targeted, and the number of cases was small, especially in women. In the future, it is necessary to consider other heart diseases such as heart failure and ischemic heart disease while considering the comorbidities of smoking, diabetes, and hypertension. Second, in this study, there were many cases with relatively good cardiac function (LVEF, VO_2_/HR, VE/VCO_2_, etc.). Examination is necessary in cases of low cardiac function. Finally, the difference with the Borg scale cannot be clearly shown. In the ROF report developed as a fatigue scale in the previous research (Micklewright et al., 2017), it is said that the scales are easy to understand because of the descriptors and diagrams. In the future, we would like to examine the difference between the RPE‐face scale and the Borg scale for more age groups.

## 5 CONCLUSION

In conclusion, we showed that there was a significant positive correlation between the RPE‐face scale and the HR, VO_2_, WR, and VE during cardiopulmonary exercise test in older adults with AF for groups, especsially individuals. For AT, the cutoff value of the RPE‐face scale was “4” and its sensitivities were from 82.8% to 96.4% and specificities 79.6% to 87.2%. These results suggest that the RPE‐face scale can be used to determine the intensity of physical exercise unaffected by gender and age, SR of AF.

## CONFLICTS OF INTEREST

None.

## AUTHOR CONTRIBUTION

SN, the primary author, oversaw writing the main parts of the manuscript, recruitment of participants, conducting the study, data collection, and statistics. SN and SM designed the research question. SN and SI conducted the testing and data collections. SN, SM, and KH analyzed the data. SM, KH, and AT contributed to the critical review of draft manuscripts and approved the final manuscript. All authors read and approved the final manuscript.

## ETHICAL APPROVAL

The study was approved by the Ethics Committee of Niigata Medical Center (Approval No. 2018–04) and the Ethics Committee of Niigata University Health and Welfare Graduate School of Medicine (Approval No. 17956–180313).
